# Can Carbodiimide (EDC) and Chitosan Cross-linking Agents Effect the Longevity of Fiberglass Posts Luted with Different Types of Composite Cements to Root Dentin?

**DOI:** 10.3290/j.jad.b3868623

**Published:** 2023-02-06

**Authors:** Helena C. Assis, Glauce C. do Nascimento, Renato Roperto, Manoel D. Sousa-Neto, Fabiane C. Lopes-Olhê

**Affiliations:** a Postgraduate Student, Department of Restorative Dentistry; School of Dentistry of Ribeirão Preto, University of São Paulo (USP), Ribeirão Preto, São Paulo, Brazil. Study conception and design, data acquisition and analysis, wrote the manuscript, approved the version to be submitted.; b Professor, Department of Basic and Oral Biology, School of Dentistry of Ribeirão Preto, University of São Paulo (USP), Ribeirão Preto, São Paulo, Brazil. Data analysis, critically revised the manuscript for important intellectual content, approved the version to be submitted.; c Professor, Department of Adult Restorative Dentistry, University of Nebraska Medical Center, UNMC College of Dentistry, Lincoln, Nebraska, USA. Study conception and design, critically revised the manuscript for important intellectual content, approved the version to be published.; d Professor, Department of Restorative Dentistry, School of Dentistry of Ribeirão Preto, University of São Paulo (USP), Ribeirão Preto, São Paulo, Brazil. Study concept and design, critically revised the manuscript for important intellectual content, approved the version to be published.; e Professor, Department of Restorative Dentistry, School of Dentistry of Ribeirão Preto, University of São Paulo (USP), Ribeirão Preto, São Paulo, Brazil. Data analysis, wrote the manuscript, interpreted data, critically revising the manuscript for important intellectual content, approved the version to be submitted.

**Keywords:** carbodiimide, chitosan, push-out, in-situ zymography, matrix metalloproteinases.

## Abstract

**Purpose:**

To evaluate the effect of carbodiimide (EDC) and chitosan (CHI) on the enzymatic activity (EA) and bond strength (BS) of different composite cements to root dentin.

**Materials and Methods:**

Ninety (90) maxillary canines were sectioned, standardizing the length of the roots. The roots were endodontically treated, prepared, divided into 3 groups according to dentin treatment (distilled water [DW], CHI 0.2 wt%, or EDC 0.5M), and further subdivided into 3 subgroups according to composite cement (RelyX ARC [3M Oral Care], Panavia F 2.0 [Kuraray Noritaki], or RelyX U200 [3M Oral Care]). Of the slices obtained by sectioning, the most cervical of each third were subjected to a push-out test and the most apical were subjected to in-situ zymography. Half of the slices were analyzed immediately, and the other half after 6 months. The results were analyzed with ANOVA or the chi-squared test.

**Results:**

RelyX ARC showed higher BS associated with CHI, while RelyX U200 showed higher BS associated with EDC (p = 0.044). For Panavia F 2.0, the treatment did not influence BS (p > 0.05). For the cervical and middle thirds, no differences were observed between the cements, while the apical third revealed higher BS for RelyX U200 (p < 0.001). The highest percentage of adhesive-to-dentin failures was observed for Panavia F 2.0. EDC showed the lowest percentage of adhesive-to-dentin failures. According to zymographic analysis, DW and CHI showed greater fluorescence for RelyX ARC, while EDC exhibited the lowest fluorescence of all cements (p > 0.05).

**Conclusion:**

The different mechanisms of action of solutions for pre-treatment of intraradicular dentin yielded different results depending on the adhesive used. EDC resulted in higher bond strength and higher enzyme inhibition for RelyX U200, while the treatment with chitosan resulted in higher bond strength and lower enzymatic activity for RelyX ARC. Although EDC and chitosan treatments did not influence the bond strength for Panavia F 2.0, both resulted in higher enzyme inhibition for this composite cement.

Fiberglass posts are commonly used for the restoration of endodontically treated teeth with extensive loss of coronal structure^48^ because their elastic modulus is similar to that of dentin. This similarity reduces the transmission of extrinsic forces to the dentin walls and consequently minimizes the risk of vertical root fractures.^44^

For adhesive cementation of posts, both conventional composite cements with chemical or dual polymerization and self-adhesive composite cements can be used. Conventional composite cements are characterized by their association with an etch-and-rinse or a self-etch adhesive, which is responsible for the bonding of the composite cement to the root dentin^23^ through the formation of a hybrid layer and intratubular tags, which result from the infiltration of resinous monomers within the exposed collagen fibrils.^29^ In contrast, self-adhesive composite cements rely on chemical adhesion to the tooth,^15^ established by a phosphoric-acid monomer, which is ionized when mixing the composite pastes, and interacts with the hydroxyapatite of the mineralized dental tissues.^7,35^

The stability of intraradicular posts is related to the integrity of the post, dentin wall, and cement interfaces.^47^ The adhesive interface with dentin is naturally susceptible to long-term degradation,^2,9,28,52^ since changes in temperature, humidity, various chemical reactions imposed by the oral environment, as well as chemical and morphological changes in the tooth structure interfere in its constitution and alter its resistance.^25^ In addition, the presence of remaining filling material on the root canal walls, high C-factor, difficult solvent evaporation, and limited light transmission to the apical root third (necessary for adequate photopolymerization) make the establishment of the adhesive interface of intraradicular posts even more complex.^3,9^ Thus, despite improvements in adhesives in recent decades, a premature decrease in bond strength, and consequent reduction in bond durability, is one of the great challenges for adhesive dentistry today.^28,44,53^

The durability of the adhesive interface is directly related to masticatory forces, water absorption, and proteolytic enzymes from the dentin itself or from exogenous sources such as bacteria^53^ and saliva, in addition to the intrinsic resistance of its constituents to degradation processes.^22^ In this sense, natural and synthetic substances with the potential to increase degradation resistance and enzymatic inhibition capacity can contribute to the maintenance of stability and longevity of the adhesive interface between composite cement and root dentin.^5^ Thus, different strategies have been proposed to minimize the degradation of the adhesive interface over time. These include the reinforcement of collagen fibers at the adhesive interface, the inhibition or inactivation of endogenous enzymes, or a combination of both strategies.^31^ In this context, synthetic substances such as carbodiimide (EDC) (1-ethyl-3- 3-dimethylaminopropyl carbodiimide hydrochloride) have demonstrated the ability to increase long-term bond strength^28^ by stimulating the formation of cross-links that already exist naturally between collagen molecules and fibrils, making dentinal collagen more resistant to degradation,^8^ in addition to having the ability to inactivate dentin metalloproteinases and cathepsin cysteines.^31^

Similarly, efforts have been made to improve the biological and mechanical properties of collagen by strengthening its matrix through the use of natural solutions. Chitosan is a hydrophilic biopolymer with a large number of free hydroxyl and amino groups.^39,50^ It is non-toxic, antifungal, antioxidant, anti-inflammatory, biocompatible, and biodegradable, and is derived from the deacetylation of chitin obtained from the exoskeleton of crustaceans; it also has the potential to increase the longevity of the bond strength of the adhesive interface to dentin.^13^ Chitosan has the ability to bind to collagen fibrils through electrostatic attraction, enveloping them, which increases collagen resistance.^38^ According to Xiong et al,^50^ the affinity between chitosan and collagenase protects collagen fibrils, blocking access to metalloproteinases and hindering their actions.

Largely, studies on the effect of natural and synthetic crosslinking agents yielded results on hybrid layer formation in coronal dentin. There are only a few studies regarding the performance of these agents in root dentin, especially when using composite cements other than etch-and-rinse, self-etching and self-adhesive composite cements.^2,4,43^ Alonso et al^2^ found that pretreatment with EDC did not weaken the initial or longer-term (9-month) bond strength of RelyX ARC (3M Oral Care) etch-and-rinse composite cement. Similarly, Ghazvehi et al^18^ observed that EDC did not decrease the bond strength of Panavia F 2.0 (Kuraray Noritake) self-etching composite cement to root dentin. However, Comba et al^9^ and Shafiei et al^43^ found higher bond strengths after 1 year for Core-X Flow (Dentsply Sirona) etch-and-rinse and Clearfil SA (Kuraray Noritake) self-adhesive composite cements, respectively. This illustrates the lack of consensus in the literature on the effect of EDC on the bond strength of different types of composite cement to root dentin. As for chitosan, the literature contains no previous studies which evaluated its effect on the bond strength and longevity of the adhesive interface with root dentin. Coronal and root dentin have different mechanical properties. Root dentin has lower hardness and lower elastic modulus compared to coronal dentin due to the different tubular density,^32^ in addition to differences in the presence and concentration of metalloproteinases (MMP) -2, -8, -9 and -13.^2^

Given the natural and synthetic substances which might increase the longevity of the adhesive interface, and due to the wide variety of composite cements with different mechanisms of adhesion to root dentin, this study evaluated the influence of EDC and chitosan treatment on the bond strength and enzymatic activity of different types of composite cements to dentin, in addition to evaluating the proteolytic activity of root dentin. The null hypotheses tested were: 1. EDC and chitosan have no effect on the bond strength of fiberglass posts cemented with different composite cements; 2. EDC and chitosan have no effect on the proteolytic activity of the dentin immediately and after 6 months; 3. aging has no effect on the bond strength and proteolytic activity.

## Materials and Methods

This study was approved by the Ethics in Research Committee of the School of Dentistry of Ribeirão Preto, University of São Paulo (CAAE: 02198018.5.0000.5419). To determine the number of samples necessary, a t-test was calculated using Sigma Plot software (Systat Software; San Jose, CA, USA). Considering a minimum difference of 1.0 MPa, a standard deviation estimate of 0.65, a statistical power of 0.9 and a probability level of α = 0.05, the estimated number was 10 specimens for each group.

Ninety maxillary canines with straight, completely formed roots, a single root canal, no calcifications, resorptions, or cracks, and a ratio of buccolingual to mesiodistal dimensions ≤ 1.5 were selected.

After sectioning the crowns and standardizing the root length to 16 mm, the root canals were instrumented with Reciproc R50 (VDW; Munich, Germany) and filled using the single-cone technique with AH Plus sealer (Dentsply Sirona; Konstanz, Germany), according to the manufacturer’s instructions.

After a period of three times the setting time, 12 mm of filling material was removed with a No. 2 Gates-Glidden drill (Dentsply Maillefer; Ballaigues, Switzerland). Then the root canals were prepared using a drill corresponding to the No. 3 Exacto fiberglass post (Angelus; Londrina, PR, Brazil). The drills were replaced after 5 post-space preparations and the 4-mm remaining filling material was confirmed by digital radiography. The prepared root canals were irrigated with 5 ml of distilled water and dried with Capillary Tip aspiration cannulas (Ultradent; South Jordan, UT, USA).

Next, the specimens were randomly divided into three groups (n = 30) according to the solution used for dentin treatment: distilled water (control), chitosan 0.2 wt%, and EDC 0.5 mol/l. Each of these was further divided into three subgroups (n = 10) according to the composite cements used for post cementation: RelyX ARC (3M Oral Care; St Paul, MN, USA), Panavia F 2.0 (Kuraray Noritake; Tokyo, Japan), and RelyX U200 (3M Oral Care) (Table 1).

**Table 1:** Composition, batch number, and manufacturer of materials used

Material	Composition	Batch number	Manufacturer
RelyX ARC	Paste A: bis-GMA, TEG-DMA, zirconia/silica, pigments, tertiary amine and photoinitiator system Paste B: bis-GMA, TED-GMA, zirconia/silica and benzoyl peroxide (filler content ≈ 67.5%)	1910700149	3M Oral Care; St Paul, MN, USA
Panavia F 2.0	Paste A: silanized silica particle, silanized colloidal silica, MDP, hydrophilic aliphatic dimethacrylate, aromatic hydrophilic dimethacrylate, camphorquinone, catalyst and initiator Paste B: silanized barium glass, sodium fluoride, aliphatic hydrophilic dimethacrylate, aromatic hydrophilic dimethacrylate and pigment catalysts (filler content ≈ 59%)	2D0227	Kuraray Noritake; Tokyo, Japan
RelyX U200	Base paste: silane-treated glass powder, 2-propenoic acid, 2-methyl 1,1‘-1- (hydroxymethyl)-1, 2-ethanodlyl ester, triethylene glycol dimethacrylate (TEG-DMA), silane-treated silica, fiberglass, sodium persulfate and per-3,5,5-trimethyl-hexanoate t-butyl Catalyst paste: silane-treated glass powder, substitute dimethacrylate, silane-treated silica, sodium p-toluenesulfonate, 1-benzyl-5-phenyl-baric acid, calcium salts, 1,12-dodecane dimethacrylate, calcium hydroxide and titanium dioxide (filler content ≈ 70%)	2000600565	3M Oral Care

The solutions were applied for 60 s using a disposable plastic syringe and NaviTip needle (Ultradent, South Jordan, UT, USA). The canals were dried with Capillary Tip cannulas and absorbent paper cones. Before cementation, the roots were wrapped with wax strips to avoid further polymerization of the cement.

For the cementation with RelyX ARC, the radicular dentin was conditioned with 35% phosphoric acid (Ultra-Etch, Ultradent, lot D0DRK) for 15 s, followed by washing with 5 ml of distilled water for 10 s. After moisture control with absorbent paper tips, the roots were treated with one of the irrigating solutions. After 60 s, the canals were again dried with absorbent paper tips. Then, Adper Single Bond 2 (3M Oral Care, lot 1922700285) etch-and-rinse adhesive was applied in two consecutive layers, followed by evaporation of the solvents using a gentle air stream for 10 s (distance of 10 cm). Excess adhesive was removed with absorbent paper cones and the material was photoactivated for 10 s using an Optilight Max photopolymerizer (Dabi Atlante; Ribeirão Preto, SP, Brazil); before use, irradiance was measured with a radiometer (L.E.D. Radiometer, Demetron Kerr; Orange, CA, USA).

For cementation with Panavia F 2.0, the canals received the dentin treatment with the tested solutions prior to applying a layer of ED Primer A (Kuraray Noritake) mixed with ED Primer B (Kuraray Noritake). The adhesives were applied in the root canals and left to act for 30 s. The adhesive excess was removed with absorbent paper cones and gentle air streams. For cementation with RelyX U200, the root canals were dried with absorbent paper cones and the material was inserted into the root canal.

The fiberglass posts were immersed in 70% alcohol for 10 min, dried, and coated with silane (Angelus; Londrina, Brazil, Lot 47285) for 60 s using a microbrush (3M Oral Care). The cements were handled according to the manufacturer’s instructions, inserted into the root canal with a size 50 K-file (Dentsply Maillefer), and applied to the fiberglass post surface, which was then inserted into the root canal using finger pressure. Photoactivation was performed for 40 s at 1200 mW/cm^2^ using an Optilight Max photopolymerizer. The specimens were stored at 37°C and 100% humidity for 7 days.

The specimens were then affixed to acrylic resin plates using hot glue. The samples were sectioned perpendicular to their long axis in the mesiodistal direction with a 0.3-mm diamond disk at 350 rpm, 75 g weight, under constant cooling using an Isomet 1000 precision cutting machine (Buehler; Lake Bluff, IL, USA). From each root third, three dentin slices 1.0 mm (±0.1 mm) thick were obtained. The first two slices of each third were used for the push-out test, and the most apical slices of each third were used for in-situ zymography. In half of the slices of each subgroup, the analyses were performed immediately, while the other half of each subgroup was stored in distilled water at 37ºC for six months before analysis.

### Push-out Bond Strength Test

Metal bases and metal stems with active tips (1.5, 1.0, and 0.8 mm) and holes (2.5, 1.5, and 1.2 mm) of different diameters, compatible with the restorative material diameter in the cervical, middle, and apical thirds, were used. The base was coupled to the lower portion of an Instron 2519-106 universal testing machine (Instron; Canton, MA, USA), and the slice was positioned with its cervical aspect facing downward in the same direction as the hole. The metal stems were attached to the upper portion of the testing machine and positioned on the intracanal restorative material. The testing machine was operated at a constant speed of 0.5 mm/min until displacement of the fiberglass post.

The force required for the displacement (F) was measured in Newtons (N). In order to calculate bond strength (BS), the resulting force was converted to MPa by dividing the force at displacement (F) by the lateral area of the intracanal material (SL). To calculate SL, the height (h) of each slice was measured using a digital caliper (Digimess Instrumentos de Precisão; São Paulo, Brazil), and the radius (major and minor) was measured by means of a stereomicroscope (Leica, M165C, Leica Microsystems; Wetzlar, Germany) using Las v4.4 software (Leica Microsystems) before the push-out tests.

For this, the following formula was used


$$\mathrm{SL}=\pi (R+r)\sqrt{h^{2}+(R-r{)}^{2}}$$

where R is the coronal radius of the restorative material, r is the apical radius of the restorative material, and h is the height/thickness of the slice.

From these data, BS was calculated in MPa by dividing the force at displacement of the fiberglass post by its lateral area (BS = F/SL).

### Mode of Failure

After the bond strength test, failure modes were determined using a Leica M165C stereomicroscope at 25X magnification. The failures were determined in percentages and classified as follows: a) adhesive to dentin, if the intracanal material detached from the dentin; (b) adhesive to composite cement, if the fiberglass post detached from the composite cement; (c) mixed, when the fiberglass post detached from both dentin and composite cement; (d) cohesive in dentin, when dentin fracture occurred; (e) cohesive in the fiberglass post, when the fiberglass post fractured; (f) mixed, when both the fiberglass post and dentin fractured.

### In-situ Zymography

In-situ zymography was performed with the objective of detecting gelatinase activity at the adhesive interface formed between radicular dentin, composite cement, and fiberglass post. Each slice was bonded to a microscope slide with cyanoacrylate glue (Loctite, Henkel; Itapevi, SP, Brazil), taking care to avoid blisters between the slice and glass surface. Then, the slices were progressively polished in a polishing machine with 400-, 600-, 1200- and 2000-grit silicon carbide papers until the final thickness was between 0.3 and 0.4 mm. The specimens were stored at 4°C for 24 h.

In-situ zymography was performed using DQ-gelatin (E12055, Molecular Probes; Eugene, OR, USA) diluted 1:10 in Tris-CaCl_2_ 50 M (pH 7.4). Twenty microliters of gelatinous solution containing fluorescein was applied on the samples, which were then incubated in a dark, humid chamber at room temperature for 1 h. Phenanthroline, an MMP inhibitor, and serine protease inhibitor PMSF, were used to confirm whether the activity at the adhesive interface was due to metalloproteases or serineproteases. After incubation, the samples were washed 3 times with phosphate buffered saline (PBS) and then fixed with 4% buffered paraformaldehyde (PFA). The samples were covered with coverslips to be examined and photographed with a Leica DMR fluorescence microscope (Leica Imaging Systems), using Leica Qwin software (Leica Imaging Systems).

**Fig 1:**
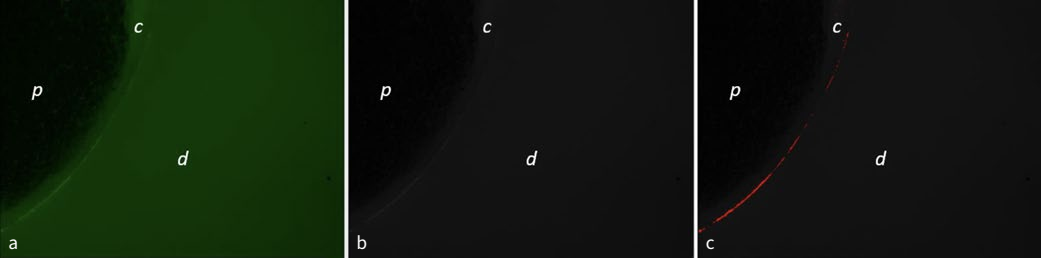
Zymographic analysis of the adhesive interface. a. RGB color image showing enzymatic activity at the adhesive interface between composite cement and root dentin; b. image converted to 8-bit grayscale; c. region of interest (ROI) delimited in red after using the threshold tool. *p:* fiberglass post; *c:* composite cement; *d:* radicular dentin.

Photomicrographs were obtained at 1.25X, 5X, and 10X magnifications, with the 5X magnification providing representative images of each quadrant of the slices. Gelatin hydrolysis, observed as the intensity of green fluorescence emitted by root dentin, was evaluated in pixels per area with ImageJ software (NIH; Bethesda, MD, USA) with an 8-bit grayscale, 23 to 255 contrast, and a threshold value of 24 to 30 (Fig 1).

### Statistical Analysis

The bond strength data were submitted to 3-way ANOVA and repeated measures ANOVA, supplemented by Tukey’s post-hoc test for multiple comparisons between groups. The possible association between the independent variables of the study (time of evaluation, dentin treatment, and cement) and the type of failure was analyzed using the chi-squared test. The proteolytic activity data were submitted to 3-way ANOVA and Tukey’s post-hoc test for multiple comparisons between groups. Statistical analyses were performed using Jamovi v.1.6.23 software (The Jamovi Project; Sydney, Australia), with a probability level fixed at 95% for all tests.

## Results

### Bond Strength Analysis

Three-way ANOVA revealed a difference for the factors cement (p < 0.001) and dentin treatment (p < 0.004), and for the interaction of cement x dentin treatment (p < 0.001). However, in terms of time of evaluation, no significant difference was found (p = 0.267).

For RelyX ARC, the greatest bond strengths were observed for chitosan vs distilled water (p = 0.003). For Panavia F 2.0, there was no difference between dentin treatments (p > 0.05). As for RelyX U200, the highest bond strength was observed for EDC compared to the other treatments (p = 0.029) (Table 2).

**Table 2:** Bond strength means ± SD in MPa for the different dentin treatments and composite cements

	Composite cement		
Dentin treatment	RelyX ARC	Panavia F 2.0	RelyX U200
Distilled water	4.4 ± 4.0^Bb^	3.9 ± 3.7^Ab^	7.2 ± 5.7^Ba^
Chitosan	8.0 ± 6.8^Aa^	2.8 ± 2.8^Ab^	7.1 ± 5.0^Ba^
EDC	6.6 ± 5.2^ABb^	4.1 ± 5.0^Ab^	10.1 ± 4.6^Aa^

Different superscript capital letters indicate significant differences between rows and different superscript lowercase letters indicate significant differences between columns (Tukey’s test, p < 0.05). n = 10, 30 slices per group, total of 540 slices.

With the distilled water treatment, the highest bond strengths were observed for RelyX U200 (p = 0.008). Using chitosan, the highest bond strengths were observed for RelyX ARC and RelyX U200 (p < 0.001). For EDC, the highest bond strengths were observed for RelyX U200 (p = 0.004) (Table 2).

Repeated-measures ANOVA revealed a significant difference for the factor root third (p = 0.022) as well as the interaction between root third and cement (p < 0.001).

For RelyX ARC and Panavia F 2.0, there was no significant difference between the root thirds (p > 0.05), while for RelyX U200, the highest bond strengths were observed for the apical third (p < 0.05) (Table 3).

**Table 3:** Bond strength means ± SD in MPa of composite cements to root dentin by root thirds

	Composite cement		
Root thirds	RelyX ARC	Panavia F 2.0	RelyX U200
Cervical	6.8 ± 6.2^Aa^	5.8 ± 4.8^Aa^	7.9 ± 3.7^ABa^
Middle	5.4 ± 4.8^Aa^	5.3 ± 4.5^Aa^	6.3 ± 4.2^Ba^
Apical	4.6 ± 5.2^Ab^	5.5 ± 5.4^Ab^	11.0 ± 7.8^Aa^

Different superscript capital letters indicate significant differences between rows and different superscript lowercase letters indicate significant differences between columns (Tukey’ test, p < 0.05). n = 10, 30 slices per group, total of 540 slices.

For the cervical and middle thirds, no significant differences were observed between the cements (p > 0.05), while for the apical third, the highest bond strengths were observed for RelyX U200 (p < 0.001) (Table 3).

### Failure-pattern Analysis

The chi-squared test showed that Panavia F 2.0 had a greater percentage of adhesive-to-dentin failure compared to RelyX ARC and RelyX U200 (p < 0.001). EDC showed a lower percentage of adhesive failures to dentin compared to chitosan and distilled water (p < 0.001; Table 4).

**Table 4:** Failure mode types after push-out test for dentin treatments and different composite cements (in %)

Composite cement	Failure type	Dentin treatment		
		Water	Chitosan	EDC
RelyX ARC	Ad	28.3	26.7	3.3
	Ap	16.7	11.7	53.3
	Ma	23.3	33.3	15.0
	Cd	18.3	10.0	15.0
	Cp	6.7	5.0	1.7
	Mc	6.7	13.3	11.7
Panavia F 2.0	Ad	61.7	41.7	16.7
	Ap	16.7	26.7	53.3
	Ma	15.0	15.0	15.0
	Cd	5.0	16.7	11.7
	Cp	0.0	0.0	1.7
	Mc	1.7	0.0	1.7
RelyX U200	Ad	38.3	30.0	10.0
	Ap	21.7	20.0	51.7
	Ma	13.3	18.3	25.0
	Cd	13.3	20.0	8.3
	Cp	6.7	3.3	1.7
	Mc	6.7	8.3	3.3

Failure type: Ad: adhesive to dentin; Ap: adhesive to post; Ma: mixed adhesive; Cd: cohesive in dentin; Cp: cohesive in post; Mc: mixed cohesive.

### Proteolytic Activity Analysis

Three-way ANOVA revealed a signficant difference for the factors cement (p < 0.001) and dentin treatment (p < 0.001), as well as for the interaction between cement x dentin treatment (p < 0.002).

For RelyX ARC, the lowest number of pixels per fluorescent area (ie, lowest fluorescence) was observed for EDC (p < 0.001). For Panavia F 2.0 and RelyX U200, the lowest fluorescence was observed with both EDC and chitosan (p < 0.001) (Table 5).

**Table 5:** Means ± SD in mm^2^ of the fluorescent area after in-situ zymography for different treatments of dentin and composite cements

	Composite cement		
Dentin treatment	RelyX ARC	Panavia F 2.0	RelyX U200
Distilled water	1.8 ± 0.8^Ab^	1.1 ± 0.6^Aa^	1.0 ± 0.5^Aa^
Chitosan	0.8 ± 0.5^Ba^	0.4 ± 0.3^Bab^	0.2 ± 0.3^Bb^
EDC	0.0 ± 0.0^Ca^	0.0 ± 0.1^Ba^	0.0 ± 0.0^Ba^

Different superscript capital letters indicate significant differences between rows and different superscript lowercase letters indicate significant differences between columns (Tukey’s test, p < 0.05). n = 2, 6 slices per group, total of 108 slices.

For distilled water, the lowest fluorescence was observed for Panavia F 2.0 and RelyX U200 (p < 0.001). For chitosan, the lowest fluorescence was observed for RelyX U200 (p = 0.008). As for EDC, no signficant differences were observed between the cements (p > 0.05) (Table 5).

Qualitative analysis of in-situ zymography images revealed no signficant difference in the adhesive interface initially or after six months. However, significant differences were observed between the dentin-treatment solutions associated with different composite cements.

**Fig 2:**
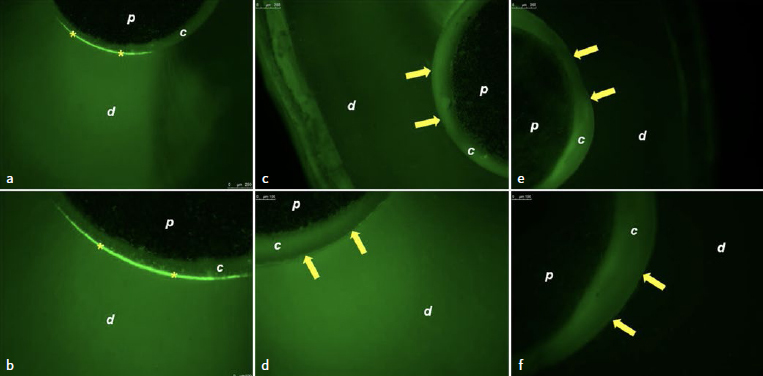
Photomicrographs of the adhesive interface of fiberglass posts cemented with RelyX ARC. a. Treatment with distilled water showing gelatinolytic activity at the adhesive interface (5X); b. treatment with distilled water showing gelatinolytic activity at the adhesive interface (10X); c. treatment with chitosan showing absence of enzyme activity (5X); d. treatment with chitosan showing absence of enzyme activity (10X); e. treatment with EDC showing absence of enzyme activity (5X); f. treatment with EDC showing absence of enzyme activity (10X). *p:* fiberglass post; *c:* composite cement; *d:* radicular dentin. Yellow asterisks: gelatinolytic activity; yellow arrows: absence of enzyme activity.

With regard to the adhesive interface of the fiberglass posts cemented with RelyX ARC, the presence of fluorescence was observed in the distilled water group, which is evidence of gelatinolytic/collagenolytic activity (Figs 2a and 2b). As for the group treated with chitosan (Figs 2c and 2d) and EDC (Figs 2e and 2f), no enzymatic activity was observed (absence of fluorescence) at the adhesive interface.

**Fig 3:**
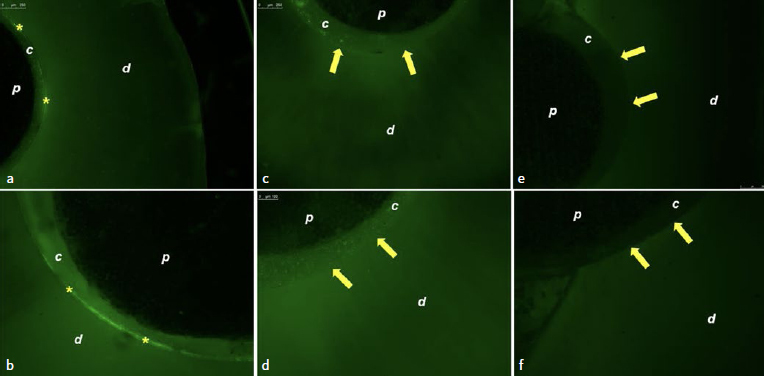
Photomicrographs of the adhesive interface of fiberglass posts cemented with Panavia F 2.0: a. Treatment with distilled water showing gelatinolytic activity at the adhesive interface (5X); b. treatment with distilled water showing gelatinolytic activity at the adhesive interface (10X); c. treatment with chitosan showing absence of enzyme activity (5X); d. treatment with chitosan showing absence of enzyme activity (10X); e. treatment with EDC showing absence of enzyme activity (5X); f. treatment with EDC showing absence of enzyme activity (10X). *p:* fiberglass post; *c:* composite cement; *d:* radicular dentin; yellow asterisks: gelatinolytic activity; yellow arrows: absence of enzyme activity.

Regarding Panavia F 2.0, for the group treated with distilled water, fluorescence was observed at the cement/dentin interface (Figs 3a and 3b). In the group treated with chitosan (Figs 3c and 3d) and EDC (Figs 3e and 3f), there was no fluorescence at the adhesive interface.

**Fig 4:**
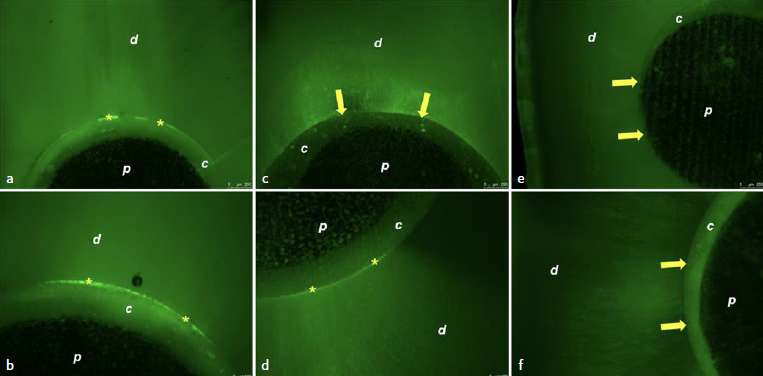
Photomicrographs of the adhesive interface of fiberglass posts cemented with RelyX U200: a. Treatment with distilled water showing gelatinolytic activity at the adhesive interface (5X); b. treatment with distilled water showing gelatinolytic activity at the adhesive interface (10X); c. treatment with chitosan showing absence of enzyme activity (5X); d. treatment with chitosan showing gelatinolytic activity at the adhesive interface (10X); e. treatment with EDC showing absence of enzyme activity (5X); f. treatment with EDC showing absence of enzyme activity (10X). *p:* fiberglass post; *c:* composite cement; *d:* radicular dentin; yellow asterisks: gelatinolytic activity; yellow arrows: absence of enzyme activity.

Regarding RelyX U200, for the group treated with distilled water (Figs 4a and 4b), fluorescence was observed at the adhesive interface. For the group treated with chitosan (Figs 4c and 4d), both absence of fluorescence and areas of gelatinolytic/collagenolytic activity were observed. In the group treated with EDC (Figs 4e and 4f), there was no fluorescence at the adhesive interface.

## Discussion

Previous studies demonstrated that long-term loss of the adhesive interface is the most common cause of restoration failure, since the bond between dentin and resin composite is constantly subjected to thermal and mechanical challenges.^26^ The characteristics of the dentinal substrate itself, such as structural and morphological heterogeneity,^27^ moisture from exudation of dentinal fluid,^12^ and collagen degradation due to collagenolytic enzymes in root dentin, promote a decrease in bond strength over time.^54^ Thus, it is important to study substances for dentin surface treatment that may increase the longevity of the bond of the different composite cements, which have different mechanisms of adhesion, to root dentin.

Regarding the methodology, maxillary canines that had a single root canal and a buccolingual-to-mesiodistal dimensional ratio ≤ 1.5^36^ were selected so that the cross section was as circular as possible,^37^ similar to the cross section of the fiberglass posts, allowing better adaptation of the adhesive interface.

To evaluate the bond strength of the restorative materials to root dentin, the push-out test was used, which makes it possible to determine the bond strength in the different thirds of the root canal.^30^ For this, bases and metallic stems with active tips and holes of compatible diameters were made for each root third, in order to facilitate the application of force and more evenly distributed shear stresses as close as possible to the composite-cement/dentin interface.^28,52^

Following the push-out test, failure patterns were analyzed under a stereomicroscope, and the proteolytic activity of the adhesive interface was analyzed using in-situ zymography. In this technique, the gelatinous substrate is degraded by active gelatinolytic enzymes present in the dentin sample, a process detectable by fluorescence microscopy. The fluorescence intensity is directly proportional to the tissue’s proteolytic activity.^2^ A protocol adapted from the study by Mazzoni et al^34^ was used to allow detection of dentinal proteases, such as matrix metalloproteinases (MMPs) and cathepsin cysteines. It is known that these enzymes are zinc and calcium dependent, regulate physiological and pathological metabolism, are released during dentin demineralization by acid conditioner or acid monomers,^31^ and are capable of degrading collagen fibers. Composite cement infiltrates the collagen-fiber mesh to form the adhesive interface^28,43^ by breaking peptide bonds.^20^

To allow evaluation of the bond strength longevity and proteolytic activity, accelerated aging was performed by direct exposure of the slices to distilled water,^9,28,52^ with the aim of leading to rapid diffusion through the adhesive interface, resulting in degradation of the dentinal collagen matrix and resin.^27,43^

The results showed that for RelyX U200 self-adhesive cement, dentin treatment with EDC resulted in higher bond strength and a lower percentage of adhesive-to-dentin failures compared to chitosan and distilled water, as well as lower enzymatic activity compared to distilled water (Figs 4e and 4f). The latter may be related to the fact that EDC establishes peptide-covalent bonds between proteins, which reduces the molecular mobility essential for the collagenolytic activity of enzymes,^33,43^ unlike chitosan, which binds to collagen fibrils electrostatically.^13^ The mechanism of protease inhibition by EDC occurs through activation of the carboxyl group of glutamic and aspartic acids present in collagen molecules and in the active site of MMPs.^40^ Thus, the low pH of etched dentin after performing the acid-etching step of RelyX ARC etch-and-rinse cement suppresses the ionization of the carboxyl group that is activated by EDC, decreasing its effectiveness.^1,42^ On the other hand, RelyX U200 cement is applied over previously unetched dentin. Thus, at neutral pH, EDC has greater stability, where the carboxylic groups are fully ionized and negatively charged,^42^ resulting in cross-links without residual reactive groups.^41^ This may explain the better performance of RelyX U200 cement associated with EDC treatment.

For RelyX ARC, dentin treatment with chitosan resulted in higher bond strength compared to EDC and lower enzymatic activity compared to distilled water (Figs 2c and 2d). This may be related to electrostatic interactions between chitosan and collagen fibrils.^38^ The chitosan interaction is influenced by the pH of the dentin environment, which in turn interferes with the ionization of chitosan amino groups.^14^ Thus, after conditioning the dentin prior to appyling RelyX ARC cement, the pH becomes acidic, so that when chitosan is applied, its amino groups are ionized, conferring a positive charge and facilitating binding to negatively charged molecules such as collagen, thus becoming a bioadhesive compound.^10^ Since the use of RelyX U200 and Panavia F 2.0 cements does not require prior acid etching, the neutral pH of the dentin surface at the time of chitosan application means that large amounts of amino groups are not ionically charged.^39^ This interferes with the formation of intermolecular bonds with dentinal collagen and decreases the mechanical properties of the organic matrix.^14^ It is important to note that the higher enzymatic activity at the adhesive interface of RelyX ARC cement in relation to other cements, especially when associated with distilled water treatment (Figs 2a and 2b), is probably related to the presence of a demineralized dentin zone not infiltrated by monomers.^21^ This favors hydrolytic degradation, mediated by the action of water, and enzymatic degradation, in which water participates by reactivating MMPs and cathepsin cysteines previously inactivated by deposition of hydroxyapatite crystals.^19^

Regarding Panavia F 2.0 self-etching cement, dentin treatment with the different solutions did not influence the bond strength. However, the pretreatment of dentin with EDC and chitosan resulted in lower enzymatic activity (Figs 3c–3f) compared to distilled water, indicating that both solutions improved the quality of the adhesive interface. Unlike RelyX ARC cement, the self-etching adhesion mechanism involves simultaneous demineralization and infiltration of resinous monomers; thus, no discrepancy is generated between the depth of demineralization by acidic monomers and penetration of composite cement, causing fewer gaps.^46^ However, self-etching systems generally contain a higher concentration of hydrophilic monomers, which increases the permeability of the hybrid layer and increases the dissociation of monomers. Hence, the use of this type of adhesive also leads to the exposure of collagen, which can be degraded hydrolytically through the activation of MMPs.^27^ Therefore, previous studies sought to insert MMP inhibitors into the composition of the self-etching adhesive.^51^ However, changes were observed in the mechanical properties of composite cement, and there was no increase in the bond longevity of the adhesive interface.^45^ Thus, up to now, the literature does not conclusively elucidate the degree to which MMPs influence self-etching adhesives. The first and second null hypotheses of this study were thus rejected.

For RelyX ARC and Panavia F 2.0 cements, the bond strengths were statistically similar in all root canal thirds, while RelyX U200 cement presented higher bond strengths in the apical third compared to the other cements, demonstrating that this material was less influenced by depth and tubular density.^24^ According to Ferrari et al,^16^ the number of dentinal tubules is greater in the cervical third of the roots, gradually decreasing in the apical direction. The lower tubular density in the apical third implies a greater amount of intertubular dentin available for chemical interaction with the cement. Thus, in the self-adhesive adhesion mechanism, the bond strength to dentin seems to be more related to the amount of available intertubular dentin than to the density of the dentinal tubules,^6,17^ which may explain the results obtained.

In-vitro research has shown that bond strengths after storage in water generally decrease in the short and long term, due to hydrolytic degradation of the components of the adhesive interface.^11^ However, the results of the push-out test showed that after six months, there was no significant decrease in the bond strengths, suggesting that accelerated aging through direct exposure of the adhesive interface to distilled water for six months was not enough to destabilize the adhesive interface and significantly decrease the bond strength in the present study. It is noteworthy that degradation is a complex process that includes the disintegration and dissolution of materials in saliva and other types of mechanical, chemical and physical events caused by occlusal force and thermal stress, as well as enzymatic attacks and effects of pH variation.^49^ The third null hypothesis was accepted.

Considering that the purpose of using cross-linking agents is to promote enzymatic inhibition and increase resistance to degradation of the adhesive interface, thus contributing to the maintenance of bond strength over time, RelyX U200 cement performed better when associated with EDC treatment. For RelyX ARC cement, dentin treatment with chitosan was shown to be beneficial in the formation of the hybrid layer between composite cement and root dentin. As for the Panavia F 2.0 cement, the treatment of dentin with the evaluated solutions did not influence the bond strengths; however, both EDC and chitosan reduced the enzymatic activity.

## Conclusions

For RelyX U200, EDC resulted in higher bond strength, lower percentage of adhesive-to-dentin failures, and higher enzyme inhibition, while for RelyX ARC, chitosan resulted in higher bond strength and lower enzymatic activity. For Panavia F 2.0 the dentin treatment did not influence the bond strength; however, EDC and chitosan resulted in lower enzymatic activity. For RelyX ARC and Panavia F 2.0, the bond strength was similar in all thirds of the root canal, while RelyX U200 showed higher bond strength in the apical third. After six months, there was no decrease in bond strength. Therefore, it is suggested that the different mechanisms of action of the solutions used for pretreatment of intraradicular dentin yield different results, depending on the type of adhesive used. More studies are needed to evaluate the bond strength and proteolytic activity for longer periods, in addition to clinical studies to verify the behavior of these materials in function and under the influence of the oral environment.

## Clinical relevance

The different mechanisms of action of the solutions used for pretreatment of intraradicular dentin result in different effects, depending on the type of composite cement used.
